# Efficacy of Plant-Based Iron and Vitamin C in Adults With Iron Deficiency Anemia: A Randomized, Double-Blind Clinical Study

**DOI:** 10.7759/cureus.95268

**Published:** 2025-10-23

**Authors:** Maheshvari N Patel, Nayan Patel, Jit Maheshvari

**Affiliations:** 1 Clinical Research, NovoBliss Research Private Limited, Ahmedabad, IND; 2 Pharmacology, Swaminarayan University, Ahmedabad, IND; 3 Clinical Research Operations, NovoBliss Research Private Limited, Ahmedabad, IND; 4 Research and Development, Orgenetics, Inc., Brea, USA

**Keywords:** amla, curry leaves, iron deficiency anemia, iron supplement, vitamin c

## Abstract

Background

Iron-deficiency anemia (IDA) is a common nutritional disorder marked by low hemoglobin and impaired oxygen transport, leading to fatigue and reduced quality of life. This study assessed the efficacy and safety of two plant-based iron formulations in adults with IDA. Plant-based iron supplements were investigated to overcome some of the shortcomings of conventional synthetic iron formulations, which are often associated with gastrointestinal intolerance, poor absorption, and oxidative stress.

Methods

A randomized, double-blind, placebo-controlled trial was conducted in 96 adults (males and non-pregnant, non-lactating females) aged 26-55 years with hemoglobin levels between 8-11 mg/dL. Participants were assigned to one of three groups for 60 days as follows: group A received 18 mg of plant-based iron from *Murraya koenigii* (Orgen-I) and 90 mg of vitamin C from *Phyllanthus emblica* (Orgen-C), group B received 18 mg of plant-based iron alone, and group C received a placebo. Hematological and biochemical markers, hemoglobin, serum iron, ferritin, transferrin, RBC count, and superoxide dismutase (SOD), were measured at baseline and post-intervention.

Results

Both treatment groups showed significant hemoglobin improvement compared to placebo (p<0.001), with greater efficacy in the combination group. Serum iron and ferritin levels increased, but not significantly (p>0.05). Transferrin levels significantly decreased (p<0.0001), RBC counts increased (p<0.05), and SOD levels improved without statistical significance (p>0.05). No adverse events occurred, and participants reported improved energy and quality of life.

Conclusions

Plant-based iron supplementation, especially with vitamin C, is shown to be effective and safe in improving hematological parameters in those with IDA. Furthermore, participants reported an improved quality of life, suggesting increased gut tolerability of plant-based iron. These findings support its use as a natural option for anemia management.

## Introduction

Iron-deficiency anemia (IDA) is a pervasive health condition affecting individuals across all age groups, presenting unique challenges in each generation [1,2]. In infants and young children, IDA is often linked to inadequate dietary iron intake or malabsorption, impairing neurodevelopment and cognitive function during critical growth phases [3,4]. Adolescents, particularly females, are vulnerable to IDA due to rapid growth spurts, menstrual blood loss, and poor dietary habits, which exacerbate iron depletion [5]. Women of reproductive age face an elevated risk of IDA due to menstruation, pregnancy, and lactation, with significant implications for maternal and fetal health, including increased risks of preterm delivery and low birth weight. In the elderly, IDA is frequently associated with chronic diseases, gastrointestinal blood loss, and reduced iron absorption, contributing to fatigue, diminished physical function, and increased susceptibility to comorbidities [6,7]. Across all generations, IDA adversely impacts quality of life and economic productivity. Despite the availability of iron supplementation, adherence and tolerability remain challenges, necessitating innovative formulations that enhance absorption and minimize side effects [8].

The comparison between natural iron supplements and synthetic iron drugs highlights key differences in efficacy, tolerability, and overall health benefits. Natural iron supplements, derived from plant-based sources, such as curry leaves (*Murraya koenigii*) and vitamin C from amla (*Phyllanthus emblica*), often contain bioavailable forms of iron along with natural cofactors, which enhance absorption and utilization [9,10].

Amla (*Phyllanthus emblica*) is widely recognized as a rich natural source of vitamin C, having a documented history in Ayurveda. Amla fruit juice can have upward of 400 mg of vitamin C per 100 mL [11]. Following a water extraction process, it is possible to concentrate the vitamin C to higher levels by removing insoluble fibers. This standardized extraction ensures a consistent and potent source of natural vitamin C for therapeutic use.

Curry leaves (*Murraya koenigii*) can be a valuable natural source of many trace minerals, including iron. Dehydrated curry leaves can contain approximately 12 mg of elemental iron per 100 g, making them a potent botanical ingredient for iron supplementation. Following a water extraction process, it is possible to concentrate the iron levels by removing insoluble fibers. This standardized extraction process supports their traditional use in managing iron-deficiency anemia and could validate their inclusion in standardized plant-based formulations aimed at improving iron-deficiency anemia [12].

When combined with vitamin C, the bioavailability of iron can be significantly enhanced. Vitamin C plays a crucial role in facilitating the absorption of non-heme iron by reducing ferric (Fe³⁺) to ferrous (Fe²⁺) form in the gastrointestinal tract, thereby improving its solubility and uptake. The synergistic effect of this combination offers a more effective approach to improving iron-deficiency anemia [13]. This clinical trial aimed to study whether using plant-based standardized formulations of vitamin C and iron can have even better bioavailability and/or tolerance.

This randomized double-blind, placebo-controlled clinical study of 96 male and non-pregnant/non-lactating female adult subjects with IDA, but otherwise healthy, aged 26-55 years, investigates the potential of a botanical extract standardized for iron as an alternative and well-tolerated source of iron supplementation. Two formulations were evaluated as follows: one containing 18 mg of plant-based iron from curry leaf extract and the other being a combination blend of 18 mg of plant-based iron from curry leaf extract, along with 90 mg of plant-based vitamin C from amla extract to enhance absorption. The inclusion of vitamin C sourced from amla (*Phyllanthus emblica*) extract is intended to optimize the bioavailability of iron by facilitating its absorption in the gastrointestinal tract. These innovative formulations offer a natural and potentially more tolerable approach to addressing IDA compared to conventional synthetic options.

## Materials and methods

Ethical conduct of the study

This trial was conducted in compliance with the ethical principles outlined in the Declaration of Helsinki, the International Council for Harmonization (ICH) Good Clinical Practice (GCP) guidelines, and the regulatory standards established by the Indian Council of Medical Research (ICMR) and the Food Safety and Standards Authority of India (FSSAI). The study protocol received ethical approval from the ACEAS-Independent Ethics Committee on June 29, 2023 (#NB230024-OI). Furthermore, the trial was prospectively registered with the Clinical Trial Registry of India (CTRI) (#CTRI/2023/10/059283) on October 30, 2023, and ClinicalTrials.gov (ID: NCT06096103).

Written informed consent was obtained from all participants, guaranteeing that they were fully briefed on the study's objectives, procedures, potential risks, and benefits. This process ensured that participants were empowered to make informed decisions regarding their involvement in the study. The intervention phase spanned 60 days, during which participants received continuous administration of the assigned investigational product. Throughout the study, adherence to ethical guidelines was maintained to safeguard participant rights, safety, and well-being.

Study design

This clinical study employed a randomized, double-blind, placebo-controlled, three-arm design to comprehensively assess the safety and efficacy of two distinct oral plant-based iron supplements - a standalone extract of curry leaves (*Murraya koenigii*), standardized to 3.6% elemental iron (as quantified by atomic absorption spectroscopy), and a combination of this curry leaf extract with an extract of Amla (*Phyllanthus emblica*), standardized to 50% vitamin C (quantified by high-performance liquid chromatography). Both treatment formulations were compared to a placebo arm to evaluate their relative effectiveness in addressing iron-deficiency anemia (IDA) among participants.

A total of 96 adult subjects aged 26-55 years with IDA, but otherwise healthy, were enrolled in the study, with the aim of achieving a balanced distribution of 32 subjects in each treatment arm using a randomized code generated by an independent biostatistician, thereby facilitating robust statistical analyses. The clinical study was initiated with the first subject's first visit (FSFV) on December 12, 2023, and was successfully completed with the last subject's last visit (LSLV) on February 9, 2024, marking the completion of the study.

The primary efficacy endpoints included changes in hemoglobin levels, red blood cell count, ferritin levels, serum iron levels and hematocrit levels, while secondary endpoints comprised transferrin saturation, transferrin levels, total iron-binding capacity, white blood cell count and superoxide dismutase (SOD), and patient-reported quality of life parameters conducted at day one (baseline), day 30, and day 60. Additionally, a comprehensive safety assessment, including the evaluation of urine parameters, was conducted throughout the study to monitor any potential adverse effects. The study employed a robust methodological framework, ensuring adherence to clinical research standards and enhancing the validity and reliability of the findings. This structured trial design facilitated a comprehensive evaluation of the investigational products’ impact on IDA management.

Inclusion and exclusion criteria

Subjects enrolled in the study had IDA, but were otherwise healthy males and non-pregnant, non-lactating females between 26 and 55 years of age at the time of providing informed consent. Eligible participants were diagnosed with mild to moderate iron-deficiency anemia, with hemoglobin levels ranging from 8 to 11 g/dL. All subjects had tested negative for hepatitis B surface antigen (HBsAg) at screening. Female participants of childbearing potential had a negative urine pregnancy test and were able to maintain stable contraceptive use, hormonal therapy, or no therapy, for at least six weeks prior to and throughout the study period. Additionally, subjects were able to continue their baseline medications and nutritional supplements without changes during the study. All participants provided written informed consent and agreed to adhere to the study procedures. Furthermore, they committed not to use any prescribed or over-the-counter iron-containing medications or supplements for the treatment of iron deficiency or anemia, except for the investigational test treatments provided during the study.

Subjects were excluded from the study if they had any other blood disorders or malignancies or if they were diagnosed with severe anemia (hemoglobin <8 g/dL). Individuals with a history of chronic illnesses, acute blood loss, or surgery within the last three to six months were also excluded. Additionally, subjects with known allergies or sensitivity to any ingredients in the test treatment were not considered for inclusion. Pregnant or breastfeeding women, as well as those planning to become pregnant during the study period, were also excluded from participation.

Intervention details

This study included three test treatments as follows: test treatment A (placebo, brown-color powder), test treatment B (blend of plant-based iron and plant-based vitamin C), and test treatment C (standalone plant-based iron). All test treatments were formulated as blue capsules containing pale brown to brown powder. Test treatment A (placebo) contained no active ingredients. Test treatment B consisted of 500 mg of curry leaf extract (Orgen-I), standardized to 3.6%, yielding 18 mg of plant-based elemental iron, along with 180 mg of amla extract (Orgen-C), standardized to 50%, yielding 90 mg of vitamin C. Test treatment C contained 500 mg of curry leaf extract (Orgen-I), standardized to 3.6%, yielding 18 mg of plant-based elemental iron. The treatments were administered orally, with one capsule taken daily after a meal and swallowed whole with 240 mL of water. All test treatments were provided by Orgenetics, Inc.

Extraction protocol

The plant-based iron is extracted from dried curry leaves through a low-heat, water-based method designed to preserve the natural integrity of the compounds found in the source plant. The method facilitates the concentration of iron while preserving its naturally occurring cofactors and bioactive compounds, maintaining the complete phytochemical matrix of the plant. The same low-heat water extraction method is used in the case of vitamin C from dried amla fruit. The extract powder is tested for heavy metals, microbiological controls, and pesticide residue using qualified methods to ensure safety.

Randomization and blinding

This study utilized a 1:1:1 randomization ratio, with participants randomly allocated to one of three treatment arms as follows: placebo, iron supplement, or iron with vitamin C. The randomization sequence was generated using R software version 4.3.1 (64-bit) (Vienna, Austria: R Foundation) by an independent biostatistician to ensure unbiased allocation and eliminate selection bias.

To uphold the double-blind design, study personnel responsible for investigational product dispensing and distribution were not involved in any other study-related activities. Additionally, participants remained blinded to their assigned treatment to mitigate performance and reporting bias. This rigorous blinding protocol was essential in preserving the integrity of outcome assessments, enhancing the reliability of the data, and minimizing potential bias associated with participant perception and investigator influence.

Study procedures and evaluations

This study followed a structured protocol comprising four key visits to ensure comprehensive participant evaluation and systematic data collection. Visit one (screening) involved an eligibility assessment, obtaining informed consent, and conducting baseline evaluations, including blood and urine sample collection. Visit two (day one) involved potential participant enrollment, followed by randomization into one of three treatment groups as follows: iron supplement, iron with vitamin C, or placebo. Baseline blood samples were collected, and participants were provided with diaries to document the product use. Visit three (day 30±2 days) included follow-up assessments of hematological parameters, quality of life (QoL) evaluations, product perception questionnaires, and adverse event (AE) monitoring. The final visit, visit four (day 60±2 days), involved end-of-study assessments, including blood and urine sample collection, QoL evaluations, and compliance verification. Key hematological parameters, including hemoglobin, hematocrit, red blood cell (RBC) count, serum iron, ferritin, transferrin, transferrin saturation, and total iron-binding capacity (TIBC), were analyzed at baseline, day 30, and day 60, while additional biomarkers, such as white blood cell (WBC) count, superoxide dismutase (SOD), and urine parameters were assessed at baseline and day 60. This structured and methodologically rigorous approach ensured a robust evaluation of the safety and efficacy of the investigated products in participants with iron-deficiency anemia (IDA).

Blood sample collection was performed at the clinical site under standardized conditions by trained personnel. All collected samples were transported under controlled conditions and analyzed at a Good Laboratory Practice (GLP)-certified central laboratory to ensure data reliability and compliance with quality standards. Following the analysis, all laboratory reports were retrieved, compiled, and subjected to further statistical evaluation for the interpretation of study outcomes.

Sample size calculation

The sample size was calculated based on the primary endpoint change in serum ferritin levels from baseline to week four. Based on prior literature, the estimated mean change is 6.7 with a standard deviation of ±11.8. To detect a statistically significant increase in ferritin levels with 85% power and a one-sided significance level of 5%, the calculated effect size was 0.568. This yielded a required sample size of 30 subjects per arm. Accounting for a 10% dropout rate, a total of 33 subjects per arm will be enrolled to ensure 30 completers per arm for final analysis. A total of 96 subjects were randomized into three treatment arms (n=32 per arm). Of these, 86 participants completed the study.

Statistical analysis

All data were reviewed before analysis to ensure accuracy and completeness. Missing data were excluded from the analysis. The results of the statistical tests, evaluated using the paired t-test and including p-values, were reported along with corresponding confidence intervals to provide measures of precision and reliability. 

Continuous variables were summarized using descriptive statistics, including sample size (n), mean, standard deviation (SD), median, minimum, and maximum values. Categorical variables were presented as frequencies and percentages, with graphical representations provided where applicable. Statistical analysis was conducted using SPSS version 29.0.1.0 (Armonk, NY: IBM Corp.) for Windows and Excel 2019 (Redmond, WA: Microsoft Corp.) applying a 5% level of significance. Subjects who were withdrawn from the study were excluded from the statistical analysis.

## Results

Demographics and other baseline characteristics

**Figure 1:**
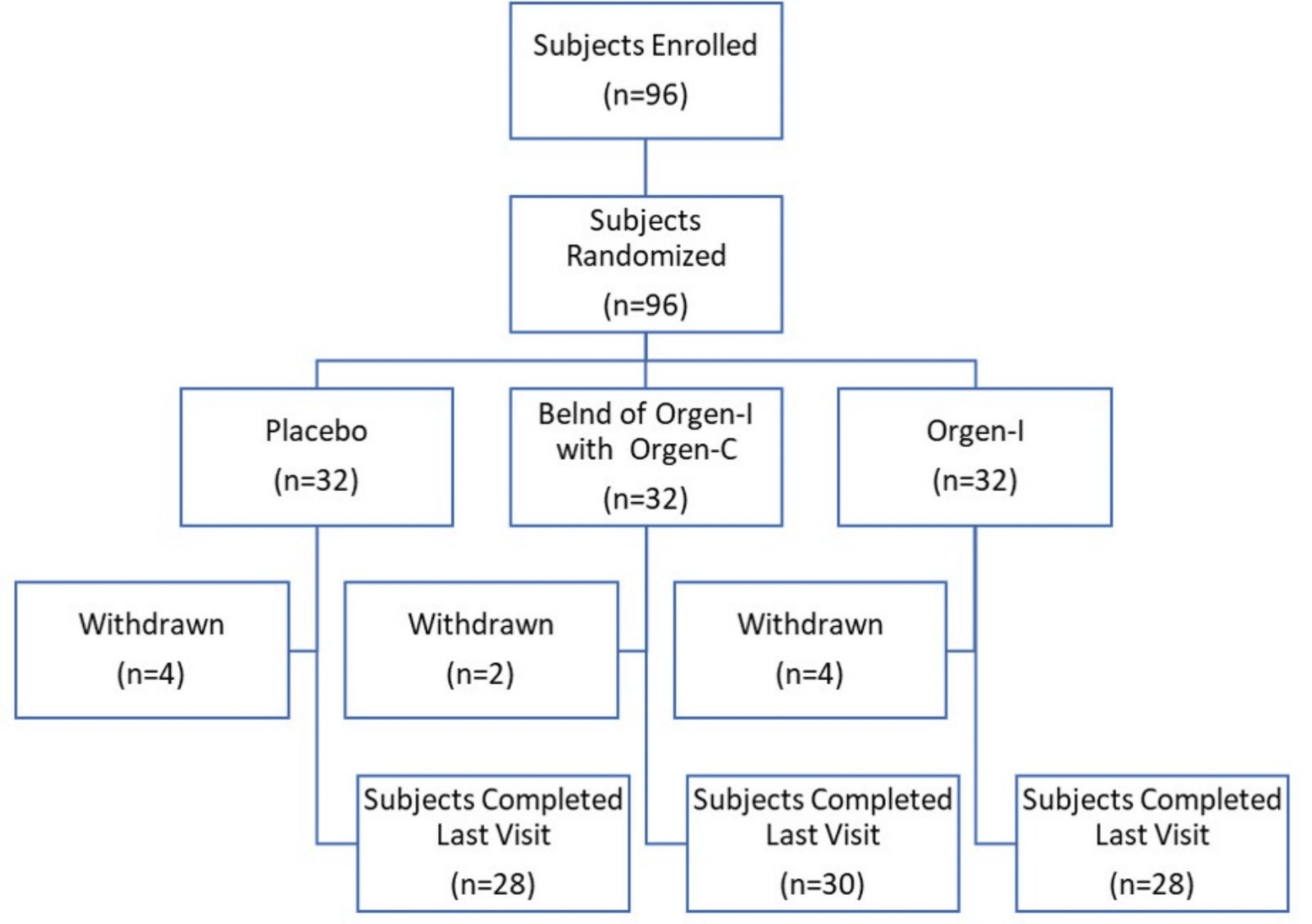
Demographic representation of patients in the placebo, Blend of Orgen-I with Orgen-C, and Orgen-I alone groups.

The study cohort included a balanced gender distribution, with 52.08% females and 47.92% males. Participants had a mean age of 38.57±6.42 years, an average height of 160.11±9.96 cm, and a mean weight of 64.07±12.80 kg. These demographic and baseline characteristics ensured a well-defined population for assessing the efficacy and safety of the iron supplementation treatments (Figure 1).

Ten subjects were withdrawn from the study due to loss to follow-up during the further subsequent evaluation visits. Consequently, these subjects were not included in the statistical analysis to maintain the integrity of the per-protocol analysis.

Primary efficacy endpoints

In the placebo group mean hemoglobin value at baseline was 10.49±0.40 g/dL, which reached 11.19±0.90 g/dL (6.80% increase, p<0.05) at day 60, while those receiving the iron supplement with vitamin C demonstrated a mean value of 10.23±0.79 g/dL at baseline, which further reached 11.11±1.71 g/dL (8.40% increase, p<0.05) at day 60. Notably, the iron supplement yielded the most pronounced improvement, with a mean value of 10.26±0.72 at baseline, which reached 11.60±1.35 g/dL (12.96% increase, p<0.0001) at day 60. These findings indicate that both standalone iron supplementation and its combination with vitamin C effectively enhanced hemoglobin levels compared with baseline, with the combination showing superior efficacy.

As per the intent-to-treat analysis, in placebo group mean hemoglobin value at baseline was 10.31±0.63 g/dL which reached to 11.19±0.90 g/dL (6.80% increase, p<0.05) at day 60, while those receiving the iron supplement with vitamin C demonstrated a mean value of 10.18±0.79 g/dL at baseline which further reached to 11.11±1.71 g/dL (8.40% increase, p<0.05) at day 60. Notably, the iron supplement yielded the most pronounced improvement, with a mean value of 10.28±0.70 g/dL at baseline, which reached 11.60±1.35 g/dL (12.96% increase, p<0.0001) at day 60.

The placebo group exhibited a mean in red blood cell (RBC) count at baseline of 4.34±0.46 million/mm^3^, which increased to 4.50±0.57 million/mm^3^ (4.15%, p>0.05) relative to baseline. Subjects receiving combination of iron and vitamin C showed a mean of 4.34±0.63 million/mm^3^, which further demonstrated a statistically significant increase of 4.54±0.66 (5.16%, p<0.05), while those administered the iron supplementation alone exhibited a mean of 4.50±0.55 million/mm^3^, which further showed a greater mean rise of 4.73±0.53 million/mm^3^ (5.96%, p<0.05). These results indicate that both standalone iron supplementation and its combination with vitamin C significantly improved RBC levels compared to placebo, with the combination therapy demonstrating a more pronounced effect.

As per the intent-to-treat analysis, the placebo group exhibited a mean in red blood cell (RBC) count at baseline of 4.29±0.48 million/mm^3^, which increased to 4.50±0.57 million/mm^3^ (4.15%, p>0.05) relative to baseline. Subjects receiving a combination of iron and vitamin C showed a mean of 4.36±0.63 million/mm^3^, which further demonstrated a statistically significant increase of 4.54±0.66 million/mm^3^ (5.16%, p<0.05), while those administered the iron supplementation alone exhibited a mean of 4.52±0.55 million/mm^3^, which further showed a greater mean rise of 4.73±0.53 million/mm^3^ (5.96%, p<0.05).

The placebo group exhibited a mean in ferritin level of 29.93±70.82 ng/mL, which increased to 30.38±60.94 ng/mL (20.74%, p>0.05), while subjects receiving a combination of iron and vitamin C exhibited a mean of 25.12±25.04 ng/mL, which demonstrated a rise of 29.25±32.22 ng/mL (46.42%, p>0.05). Notably, those receiving iron supplementation alone exhibited a mean of 31.93±37.54, which further showed a greater increase of 37.20±43.39 ng/mL (40.79%, p>0.05). Although the differences did not reach statistical significance, both iron supplementation regimens resulted in numerically higher ferritin levels compared to placebo, suggesting a potential trend toward enhanced iron storage with supplementation.

**Figure 2:**
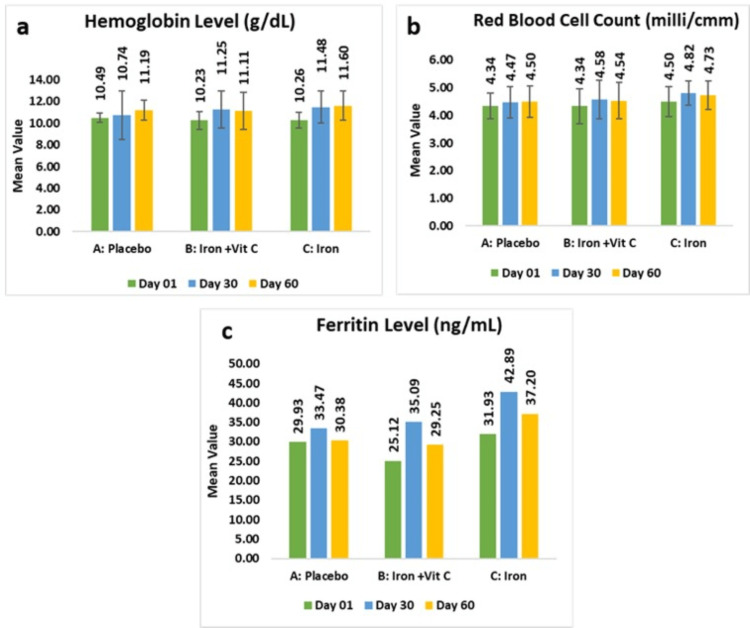
Change in hemoglobin levels (a), red blood cell counts (b), and ferritin levels (c) as per PPA. PPA: per-protocol analysis

As per the intent-to-treat analysis, placebo group exhibited a mean in ferritin level of 28.68±66.80 ng/mL, which increased to 30.38±60.94 ng/mL (20.74%, p>0.05), while subjects receiving the combination of iron and vitamin C exhibited mean of 24.06±25.58 ng/mL, which demonstrated a rise of 29.25±32.22 ng/mL (46.42%, p>0.05). Notably, those receiving the iron supplementation alone exhibited a mean of 66.92±136.13, which further showed a greater increase of 37.20±43.39 ng/mL (31.67%, p>0.05) (Figures 2a-2c).

The placebo group exhibited a mean hematocrit level of 32.17±4.59%, which increased to 35.56±3.80% (15.21%, p<0.05) from baseline. Subjects receiving the combination of iron and vitamin C exhibited a mean of 32.78±3.06%, which showed a statistically significant increase to 34.65±5.02% (6.04%, p<0.05), while those supplemented with the iron supplement alone showed a mean of 33.04±2.74%, which experienced a more pronounced elevation to 36.63±3.99% (11.34%, p<0.005). These findings suggest that both iron supplementation and its combination with vitamin C effectively enhance hematocrit levels, with the combination therapy demonstrating the greatest improvement.

As per the intent-to-treat analysis, the placebo group exhibited a mean hematocrit level of 32.17±4.59%, which increased to 35.56±3.80% (15.75%, p<0.05) from baseline. Subjects receiving the combination of iron and vitamin C exhibited a mean of 32.78±3.06%, which showed a statistically significant increase to 34.65±5.02% (6.04%, p<0.05), while those supplemented with the iron supplement alone showed a mean of 33.04±2.74%, which experienced a more pronounced elevation to 36.63±3.99% (11.34%, p<0.005).

The placebo group exhibited a mean serum iron level of 65.86±91.20 µg/dL at baseline, which further rose to 63.07±28.00 µg/dL (51.83%, p>0.05) from baseline. Subjects receiving the iron with vitamin C supplementation exhibited 46.93±24.64 µg/dL at baseline, which demonstrated a statistically significant increase of 77.35±69.36 µg/dL (82.85%, p<0.05), whereas those receiving the iron alone showed a mean of 48.21±25.12 µg/dL at baseline and showed a non-significant elevation of 59.64±35.06 µg/dL (43.52%, p>0.05). These findings suggest that while iron supplementation alone may contribute to an improvement in serum iron, the combination with vitamin C enhances iron absorption more effectively.

**Figure 3:**
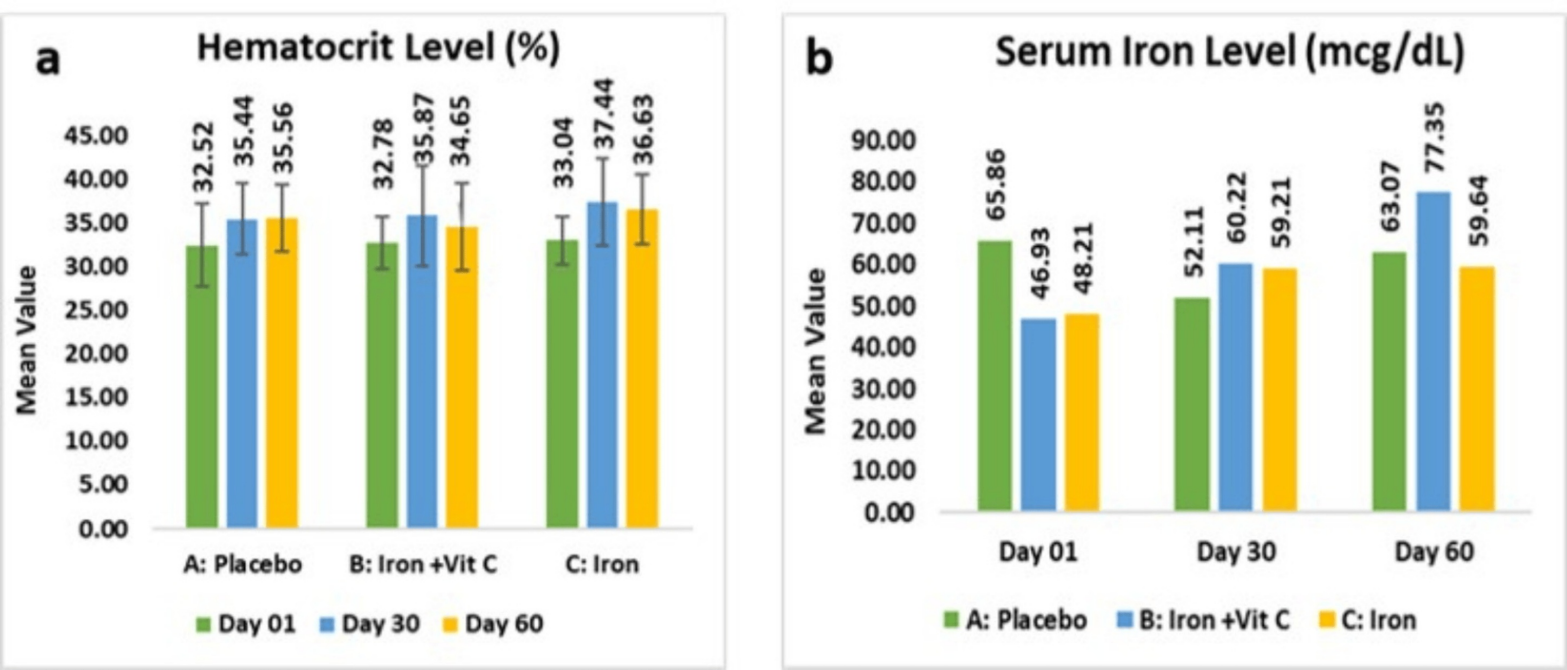
Change in hematocrit level (a) and serum iron level (b).

The placebo group exhibited a mean serum iron level of 65.50±88.25 µg/dL at baseline, which further rose to 63.07±28.00 µg/dL (51.83%, p>0.05) from baseline. Subjects receiving the iron with vitamin C supplementation exhibited 44.63±25.17 µg/dL at baseline which demonstrated a statistically significant increase of 77.35±69.36 µg/dL (110.99%, p<0.05), whereas those receiving the iron alone showed mean of 61.48±66.18 µg/dL at baseline showed a non-significant elevation of 59.64±35.06 µg/dL (47.32%, p>0.05) (Figures 3a, 3b). 

Secondary efficacy endpoints

The placebo exhibited a mean transferrin level of 2.74±0.86 g/dL at baseline, which demonstrated a significant increase of 3.23±0.79 g/dL (31.44%, p<0.05) from baseline. Conversely, subjects receiving the iron and vitamin C combination showed 2.37±0.59 g/dL at baseline, which further demonstrated a statistically significant reduction of 1.65±0.36 g/dL (-28.40%, p<0.0001), while those receiving the iron supplementation alone showed a mean of 2.02±0.63 g/dL and experienced a decrease of 1.57±0.39 g/dL (-20.04%, p<0.0001). These findings indicate that both iron supplementation regimens effectively reduced transferrin levels, suggesting enhanced iron absorption and improved iron homeostasis compared to placebo.

As per the intent-to-treat analysis, the placebo exhibited a mean transferrin level of 2.68±0.84 g/dL at baseline, which demonstrated a significant increase of 3.23±0.79 g/dL (31.44%, p<0.05) from baseline. Conversely, subjects receiving the iron and vitamin C combination showed 2.34±0.61 g/dL at baseline, which further demonstrated a statistically significant reduction of 3.06±0.68 g/dL (-31.91%, p<0.0001), while those receiving the iron supplementation alone showed a mean of 2.02±0.61 g/dL and experienced a decrease of 2.98±0.74 g/dL (-20.04%, p<0.0001).

The placebo group had a mean transferrin saturation of 10.35±7.32% at baseline and demonstrated a marked rise of 19.51±14.78% (105.03%, p<0.001) from baseline. Subjects receiving the iron and vitamin C combination exhibited an 11.20±7.45% statistically significant increase, reaching 19.37±18.58% (138.35%, p<0.05), whereas those receiving iron supplementation alone showed a 14.76±18.65% increase, with a modest, non-significant rise to 16.85±15.41% (61.00%, p>0.05). These results suggest that both iron supplementation regimens contributed to improved transferrin saturation, with iron alone exhibiting the most pronounced effect.

As per the intent-to-treat analysis, the placebo group had a mean transferrin saturation of 15.01±20.58% at baseline and demonstrated a marked rise of 19.51±14.78% (98.87%, p>0.05) from baseline. Subjects receiving the iron and vitamin C combination exhibited a 10.79±7.39% statistically significant increase to 19.37±18.58% (138.35%, p<0.05), whereas those receiving iron supplementation alone showed a 14.60±17.79% increase, with a modest, non-significant rise to 16.85±15.41% (61.00%, p>0.05).

The placebo group showed a mean in total iron-binding capacity of 462.43±120.53 µg/dL at baseline which further decreased to 434.25±114.56 µg/dL (-4.77%, p>0.05), while subjects receiving combination of iron and vitamin C exhibited mean of 437.17±69.85 µg/dL at baseline, which showed a minimal change of 434.23±81.50 µg/dL (-0.22%, p>0.05). Similarly, those receiving the iron supplementation showed 457.82±83.30 µg/dL at baseline, which further decreased to 437.50±120.23 µg/dL (-4.41%, p>0.05). These findings indicate that total iron-binding capacity (TIBC) levels remained stable across all treatment groups, with no significant alterations observed following supplementation.

**Figure 4:**
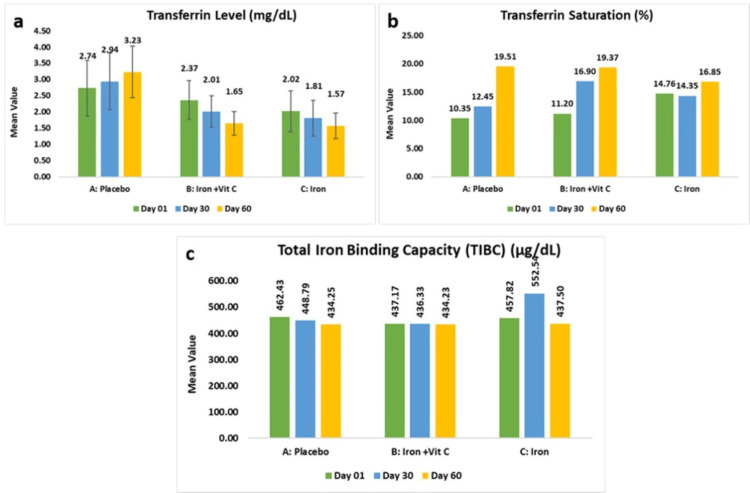
Change in transferrin level (a), transferrin saturation (b), and total iron-binding capacity (c).

As per the intent-to-treat analysis, the placebo group showed a mean in total iron-binding capacity of 465.38±114.58 µg/dL at baseline, which further decreased to 434.25±114.56 µg/dL (-4.77%, p>0.05), while subjects receiving a combination of iron and vitamin C exhibited a mean of 445.56±75.46 µg/dL at baseline, which showed a minimal change of 434.23±81.50 µg/dL (-0.22%, p>0.05). Similarly, those receiving the iron supplementation showed 458.61±80.07 µg/dL at baseline, which further decreased to 437.50±120.23 µg/dL (-4.41%, p>0.05) (Figures 4a-4c).

The placebo group exhibited a mean in white blood cell count of 7529.29±1634.41 cells/µL, which demonstrated a mean decrease of 428.93±1820.01 cells/µL (7.74%, p>0.05), while subjects receiving iron and vitamin C combination exhibited a mean of 7500.00±1630.69 cells/µL showed a larger but non-significant reduction of 3478.67±16377.85 cells/µL (50.11%, p>0.05). Conversely, those receiving the iron supplementation alone showed 8634.29±1963.18 cells/µL experienced a decrease of 559.29±2728.55 cells/µL (-3.71%, p>0.05). These findings indicate that iron supplementation, either alone or in combination with vitamin C, did not significantly influence WBC levels.

As per the intent-to-treat analysis, the placebo group exhibited mean in white blood cell count of 7513.13±1569.91 cells/µL which demonstrated a mean decrease of 428.93±1820.01 cells/µL (7.74%, p>0.05), while subjects receiving iron and vitamin C combination exhibited mean of 7543.00±1683.610 cells/µL showed a larger but non-significant reduction of 3478.67±16377.85 cells/µL (50.11%, p>0.05). Conversely, those receiving the iron supplementation alone showed 8620.65±1893.18 cells/µL experienced a decrease of 559.29±2728.55 cells/µL (-3.71%, p>0.05).

In this study, superoxide dismutase (SOD) levels were evaluated to assess the antioxidant response in participants receiving supplementation. SOD, a key enzyme in mitigating oxidative stress, plays a vital role in maintaining red blood cell integrity and supporting hemoglobin function. The analysis of SOD levels aimed to determine the potential impact of iron supplementation on oxidative balance and its association with hemoglobin synthesis. The placebo group exhibited a mean SOD of 17.77±6.16 U/mL at baseline, which slightly decreased to 15.64±4.71 U/mL (-1.36%, p>0.05), while subjects receiving the combination of iron and vitamin C showed 14.05±4.07 U/mL at baseline, which showed a modest increase of 14.58±4.23 U/mL (10.55%, p>0.05). Those supplemented with iron supplementation alone exhibited 16.14±5.88 U/mL and experienced a slightly greater rise of 17.00±4.53 U/mL (16.03%, p>0.05). Although both iron supplementation and its combination with vitamin C were associated with a numerical increase in SOD levels, the changes did not reach statistical significance.

**Figure 5:**
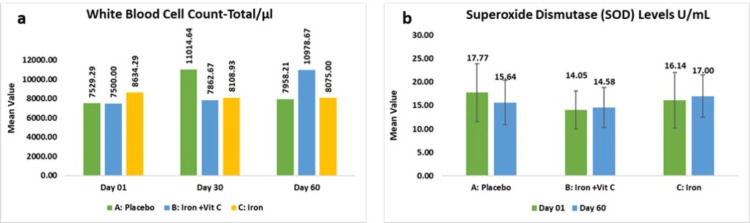
Change in white blood cell count (a) and superoxide dismutase level (b).

The placebo group exhibited a mean SOD of 17.63±6.59 U/mL at baseline, which slightly decreased to 15.64±4.71 U/mL (-1.36%, p>0.05), while subjects receiving the combination of iron and vitamin C showed 13.49±4.09 U/mL at baseline, which showed a modest increase to 14.58±4.23 U/mL (10.55%, p>0.05). Those supplemented with iron supplementation alone exhibited 15.41±6.33 U/mL and experienced a slightly greater rise of 17.00±4.53 U/mL (16.03%, p>0.05) (Figures 5a, 5b). 

Change in quality of life (QoL) product perception

Quality of life (QoL) assessments at visit 4 revealed significant improvements in energy levels, fatigue, gastrointestinal comfort, and mood among participants receiving iron supplementation. In terms of energy levels, 86.67% of participants in the iron and vitamin C blend group reported high energy levels (scores: 7-9), compared to 96.43% in the iron-only group, while only 50% of the placebo group reported similar energy levels. Regarding fatigue, 92.85% of participants receiving iron felt moderately to extremely energetic, slightly exceeding the 86.67% reported in the iron-vitamin C blend group and 67.85% in the placebo group. Gastrointestinal comfort was also notably improved, with 100% of participants receiving either iron alone or the iron-vitamin C blend reporting moderate to extreme comfort regarding nausea and gastrointestinal discomfort, compared to 75% in the placebo group. Similarly, 93.33% of those in the iron-vitamin C blend group reported being moderately to extremely comfortable with respect to constipation, slightly higher than 89.29% in the iron-only group and 67.86% in the placebo group. In terms of mood, 93.33% of participants in the iron-vitamin C blend group and 96.42% in the iron-only group reported feeling moderately to extremely joyful, compared to 85.72% in the placebo group. These findings indicate that iron supplementation, particularly iron alone, led to higher energy levels and reduced fatigue, while the iron-vitamin C blend provided comparable benefits with enhanced gastrointestinal comfort.

Comparison of test treatments with placebo

A comparative analysis was conducted to evaluate the effects of the test treatments vs. placebo across all clinical and biochemical parameters measured during the study (Table 1).

**Table 1:** Test treatment vs. placebo. SOD: superoxide dismutase; TIBC: total iron-binding capacity; CFB: change from baseline

Parameters	Treatment A vs. treatment B				Treatment A vs. treatment C			
	% CFB	p-Value	% CFB	p-Value	% CFB	p-Value	% CFB	p-Value
	Visit 3	Visit 3	Visit 4	Visit 4	Visit 3	Visit 3	Visit 4	Visit 4
Primary endpoints								
Hemoglobin (g/dL)	299.62	>0.05	24.76	>0.05	377.46	<0.05	91.33	<0.05
RBC count (×10⁶/µL)	85.64	>0.05	21.83	>0.05	140.55	>0.05	42.92	>0.05
Ferritin (ng/mL)	181.79	>0.05	817.00	>0.05	209.48	>0.05	1068.73	>0.05
Hematocrit (%)	5.88	>0.05	38.62	>0.05	50.49	>0.05	17.94	>0.05
Serum iron (µg/dL)	284.48	>0.05	1231.37	>0.05	180.00	>0.05	510.26	>0.05
Secondary endpoints								
Transferrin (g/dL)	20.96	<0.005	30.32	<0.0001	28.79	<0.0005	33.79	<0.0001
Transferrin saturation (%)	296.51	>0.05	96.29	>0.05	85.71	>0.05	49.92	>0.05
TIBC (µg/dL)	93.89	>0.05	89.59	>0.05	794.24	>0.05	10.14	>0.05
WBC count (×10³/µL)	89.59	>0.05	711.01	>0.05	115.07	>0.05	230.39	>0.05
SOD (U/mL)	-	-	125.21	>0.05	-	-	140.47	>0.05

Safety analysis

This study demonstrated a favorable safety profile for both the iron-only and iron-vitamin C blend treatments. Complete blood count (CBC) parameters, including hemoglobin, red and white blood cell counts, and platelets, remained within normal range throughout the study, with no deviations observed at screening, baseline (day one), or post-treatment assessments (days 30 and 60). Similarly, measures of iron status, such as serum iron levels, showed no abnormal variations, indicating that supplementation did not adversely affect iron metabolism. Biochemical parameters, including cholesterol, triglycerides, blood glucose, creatinine, uric acid, serum glutamate pyruvate transaminase (SGPT), serum glutamate oxaloacetate transaminase (SGOT), low-density lipoprotein (LDL), and high-density lipoprotein (HDL), remained stable across all time points, as did urine parameters related to chemical and physical characteristics. These findings confirm that both the iron-only and iron-vitamin C blend treatments were well-tolerated and did not induce any significant adverse effects in the study population.

## Discussion

The findings of this study demonstrate that both plant-based iron supplementation alone and in combination with plant-based vitamin C significantly improved key hematological parameters in individuals with iron-deficiency anemia. Compared to baseline, participants receiving the iron-vitamin C blend exhibited the highest increase in hemoglobin levels, followed by those receiving plant-based iron alone, both of which were statistically significant compared to the placebo. Similarly, red blood cell (RBC) count and hematocrit levels were notably elevated in both treatment groups, suggesting enhanced erythropoiesis and improved oxygen-carrying capacity.

Significant effect on serum iron and ferritin levels

Serum iron levels showed a significant increase in the iron-vitamin C blend group, reinforcing the role of vitamin C in enhancing iron absorption. While ferritin levels increased in both treatment groups, the changes were not statistically significant, possibly due to variations in iron storage regulation across individuals. This aligns with findings from other studies, which suggest that higher ferritin levels correlate with effective iron supplementation strategies [14,15].

Significant effect on transferrin levels

Transferrin levels significantly decreased in both treatment groups, suggesting improved iron utilization, while transferrin saturation increased, further confirming enhanced iron bioavailability. The inverse relationship between transferrin and serum iron levels is expected, as increased serum iron leads to a reduced demand for transferrin, underscoring the effectiveness of iron supplementation in improving iron dynamics [16,17]. However, the changes in total iron-binding capacity (TIBC) were not statistically significant across groups, indicating that iron homeostasis remained stable despite supplementation.

Enhanced absorption

These results align with the existing literature highlighting the role of vitamin C in enhancing non-heme iron absorption and optimizing iron metabolism. This study's strengths include its randomized, double-blind design, comprehensive safety evaluations, and assessment of both physiological and subjective outcomes. However, the relatively short study duration and sample size may limit the generalizability of the findings, warranting further long-term investigations. Several studies demonstrate that dietary intervention with iron and vitamin C effectively improves iron status and prevents iron-deficiency anemia in young women. Our findings align with this evidence, showing that separate supplementation of iron and vitamin C enhances key hematological parameters. Vitamin C aids iron absorption by converting ferric iron (Fe³⁺) to its more soluble ferrous form (Fe²⁺), improving bioavailability. Notably, individuals with better baseline iron status and lower vitamin C intake showed greater benefits, highlighting the need for personalized nutritional strategies. Future research should explore the long-term effects of supplementation and the influence of individual dietary habits on iron metabolism [18].

Several studies demonstrate that vitamin C plays a crucial role in enhancing iron absorption and improving hemoglobin levels. In our study, although significant differences in hemoglobin levels between groups have been reported in prior literature, no statistically significant difference was observed between the intervention and control groups. Dietary intake data, collected through a survey, indicated that participants in the control group consumed sufficient vitamin C from their daily diet, with an average intake meeting the recommended dietary allowance (RDA) of 90 mg, primarily from vegetables and other dietary sources. This adequate baseline intake likely contributed to similar increases in hemoglobin levels between the two groups. These findings emphasize the influence of habitual dietary vitamin C on iron status and underscore the importance of considering baseline nutrient intake when interpreting intervention effects [19].

Several studies demonstrate that oral iron supplementation effectively improves hemoglobin levels and iron stores in patients with iron-deficiency anemia (IDA), with or without additional vitamin C. The findings of this equivalence randomized controlled trial (RCT) indicate that iron supplementation alone was comparable to iron combined with vitamin C in enhancing hemoglobin levels and iron status. These results suggest that vitamin C may not be essential for improving iron absorption in individuals with IDA, challenging the conventional practice of co-administering vitamin C with iron. This highlights the need for further research to reassess the necessity of vitamin C supplementation in iron therapy protocols [20].

Safety analysis

The safety analysis confirmed that both treatment formulations were well-tolerated, with no clinically significant deviations in complete blood count, biochemical markers, or urine parameters. Importantly, the iron-vitamin C blend showed better gastrointestinal tolerability, with fewer reports of nausea and constipation compared to the placebo. Furthermore, quality of life assessments indicated improvements in energy levels, fatigue reduction, and overall well-being in the supplemented groups, with the plant-based iron-vitamin C blend demonstrating comparable or superior benefits to plant-based iron alone.

Limitations and directions for future research

This study provides valuable insights into the effects of iron and vitamin C supplementation on iron status; however, certain aspects warrant further exploration. The 60-day study duration was sufficient to observe significant changes, but a longer follow-up could provide additional insights into the sustained impact of supplementation. While dietary intake was not strictly controlled, the real-world setting enhances the applicability of our findings. Individual variations in iron absorption and metabolism highlight the need for personalized approaches in future research. Additionally, expanding the study population to diverse demographic groups would further strengthen the generalizability of the results. Future studies with extended follow-up and broader participant inclusion can build upon these findings.

## Conclusions

This study demonstrated that plant-based iron supplementation, both alone and in combination with plant-based vitamin C, effectively enhanced key hematological parameters in individuals with iron-deficiency anemia. Notably, the plant-based iron-vitamin C combination resulted in the most significant improvements in hemoglobin levels and serum iron concentrations, underscoring the role of vitamin C in facilitating iron absorption and bioavailability. Furthermore, the observed increases in RBC count, hematocrit levels, and transferrin saturation further support the clinical efficacy of both supplementation approaches in correcting iron deficiency.

Both treatment regimens were well-tolerated, with no significant adverse effects on biochemical or hematological safety markers. The plant-based iron-vitamin C combination also demonstrated superior gastrointestinal tolerability compared to iron monotherapy, contributing to enhanced patient well-being, increased energy levels, and reduced fatigue. These findings highlight the therapeutic value of plant-based iron supplementation, particularly in combination with vitamin C, for the management of iron-deficiency anemia. Future large-scale, long-term studies are warranted to confirm these findings and further assess the sustained efficacy and safety of various iron supplementation strategies.
